# Electrical impedance tomography provides information of brain injury during total aortic arch replacement through its correlation with relative difference of neurological biomarkers

**DOI:** 10.1038/s41598-024-65203-0

**Published:** 2024-06-20

**Authors:** Yitong Guo, Chen Yang, Wenjing Zhu, Rong Zhao, Kai Ren, Weixun Duan, Jincheng Liu, Jing Ma, Xiuming Chen, Benyuan Liu, Canhua Xu, Zhenxiao Jin, Xuetao Shi

**Affiliations:** 1 Shaanxi Provincial Key Laboratory of Bioelectromagnetic Detection and Intelligent Perception, Department of Biomedical Engineering, Fourth Military Medical University, Xi’an, 710032 China; 2 Department of Cardiovascular Surgery, Xijing Hospital, Fourth Military Medical University, Xi’an, 710032 China; 3 Institute of Medical Research, Northwestern Polytechnical University, Xi’an, 710072 China; 4 Department of Ultrasound Diagnosis, Tangdu Hospital, Fourth Medical University, Xi’an, 710038 China; 5 UTRON Technology Co., Ltd., Hangzhou, 310051 China

**Keywords:** Biomedical engineering, Electrical and electronic engineering, Brain imaging

## Abstract

Postoperative neurological dysfunction (PND) is one of the most common complications after a total aortic arch replacement (TAAR). Electrical impedance tomography (EIT) monitoring of cerebral hypoxia injury during TAAR is a promising technique for preventing the occurrence of PND. This study aimed to explore the feasibility of electrical impedance tomography (EIT) for warning of potential brain injury during total aortic arch replacement (TAAR) through building the correlation between EIT extracted parameters and variation of neurological biomarkers in serum. Patients with Stanford type A aortic dissection and requiring TAAR who were admitted between December 2021 to March 2022 were included. A 16-electrode EIT system was adopted to monitor each patient’s cerebral impedance intraoperatively. Five parameters of EIT signals regarding to the hypothermic circulatory arrest (HCA) period were extracted. Meanwhile, concentration of four neurological biomarkers in serum were measured regarding to time before and right after surgery, 12 h, 24 h and 48 h after surgery. The correlation between EIT parameters and variation of serum biomarkers were analyzed. A total of 57 TAAR patients were recruited. The correlation between EIT parameters and variation of biomarkers were stronger for patients with postoperative neurological dysfunction (PND(+)) than those without postoperative neurological dysfunction (PND(−)) in general. Particularly, variation of S100B after surgery had significantly moderate correlation with two parameters regarding to the difference of impedance between left and right brain which were MRAI_abs_ and TRAI_abs_ (0.500 and 0.485 with p < 0.05, respectively). In addition, significantly strong correlations were seen between variation of S100B at 24 h and the difference of average resistivity value before and after HCA phase (ΔARV_HCA_), the slope of electrical impedance during HCA (k_HCA_) and MRAI_abs_ (0.758, 0.758 and 0.743 with p < 0.05, respectively) for patients with abnormal S100B level before surgery. Strong correlations were seen between variation of TAU after surgery and ΔARV_HCA_, k_HCA_ and the time integral of electrical impedance for half flow of perfusion (TARV_HP_) (0.770, 0.794 and 0.818 with p < 0.01, respectively) for patients with abnormal TAU level before surgery. Another two significantly moderate correlations were found between TRAI_abs_ and variation of GFAP at 12 h and 24 h (0.521 and 0.521 with p < 0.05, respectively) for patients with a normal GFAP serum level before surgery. The correlations between EIT parameters and serum level of neurological biomarkers were significant in patients with PND, especially for MRAI_abs_ and TRAI_abs_, indicating that EIT may become a powerful assistant for providing a real-time warning of brain injury during TAAR from physiological perspective and useful guidance for intensive care units.

## Introduction

Total aortic arch replacement (TAAR) presents formidable challenges, as it requires circulatory arrest which may result in severe injury to central nervous system due to factors such as ischemia, hypoxia and reperfusion^1^. Even though brain protection strategies such as hypothermic circulatory arrest (HCA) and selective antegrade cerebral perfusion have been adopted during surgery, postoperative neurological dysfunction (PND) is associated with increased risk of poor prognosis even mortality for patients underwent TAAR^2,3^. In particular, stroke, delirium, paresthesia and manic-depressive psychosis are common clinical manifestations, and these permanent neurological complications significantly decrease the quality of postoperative recovery^1–3^.

Intraoperative cerebral monitoring plays an important role in the issue of improving the prognosis of patients underwent TAAR^4,5^. Existing techniques, including electroencephalogram (EEG), transcranial doppler sonography (TCD) and near-infrared spectroscopy (NIRS), have the ability of recognizing the crucial change of the physiological state of brain^4,5^. However, the evidence for the utility of EEG during operation is still at controversial^6,7^. In addition, measurements only on blood velocity from the main cerebral arteries by TCD or the cerebral oxygen saturation detected on the surface layer of the cortex using NIRS cannot be taken as convincible predictors for PND due to the consensus of most researchers that the etiology of PND are multi-factorial^4,8–12^.

Electrical impedance tomography (EIT) estimates the electrical properties at the interior of an object in a noninvasive, real-time and continuous manner^13^. Previous studies have shown that EIT were capable of monitoring the evolving process of cerebral edema by building a close negative correlation with intracranial pressure^14,15^. After our research group demonstrated that the cerebral impedance changes during HCA phase closely corresponded to PND of patients underwent TAAR^16^, we have been devoted ourselves to further study the correlation between information extracted from intraoperative EIT and physiological indicators. We realized that the PND is a series of clinical symptoms which are results from physiological and pathological change, and the clinical diagnosis of PND, especially transient PND, may be subjected to the subjective judgment of psychiatrists^17^. Therefore, in order to clarify the practicability of EIT from a more objective and statistical point of view, the relationship between EIT information and physiological indices related to PND should be investigated.

Recently, there has been renewed interest in neurological biomarkers with the hope that they would meet the needs for prediction of neurological deficits^18^. Studies have shown that biomarkers of the central nervous system (CNS) injury arise after neurons and glial cells damage produced during cardiac surgery. These biomarkers can be detected in the bloodstream, due to their diffusion across the blood–brain barrier (BBB) or the disruption of the BBB allowing these substances enter into the bloodstream^19–22^. Among these biomarkers, S100B protein, the neuron-specific enolase (NSE), glial fibrillary acidic protein (GFAP) and TAU protein drove a great interest in the field of cardiac surgery related cerebral injury^18,22,23^. A considerable amount of literature has been published on these biomarkers in their prediction of PND after cardiac surgery and the sensitivity and specificity were investigated and identified^18^.

Therefore, in this study, we investigated the correlation between intraoperative EIT extracted information and the relative difference of four specific serum biomarkers before and after operation. We hypothesized that the EIT extracted parameters from patients with PND would have strong correlation with the changes of neurological biomarkers in serum. Based on the results of our research, we believed that, from physiological perspective view, intraoperative EIT could provide auxiliary information for an early warning of brain injury during TAAR surgery.

## Results

### Patient demographic and clinical characteristics

**Figure 1:**
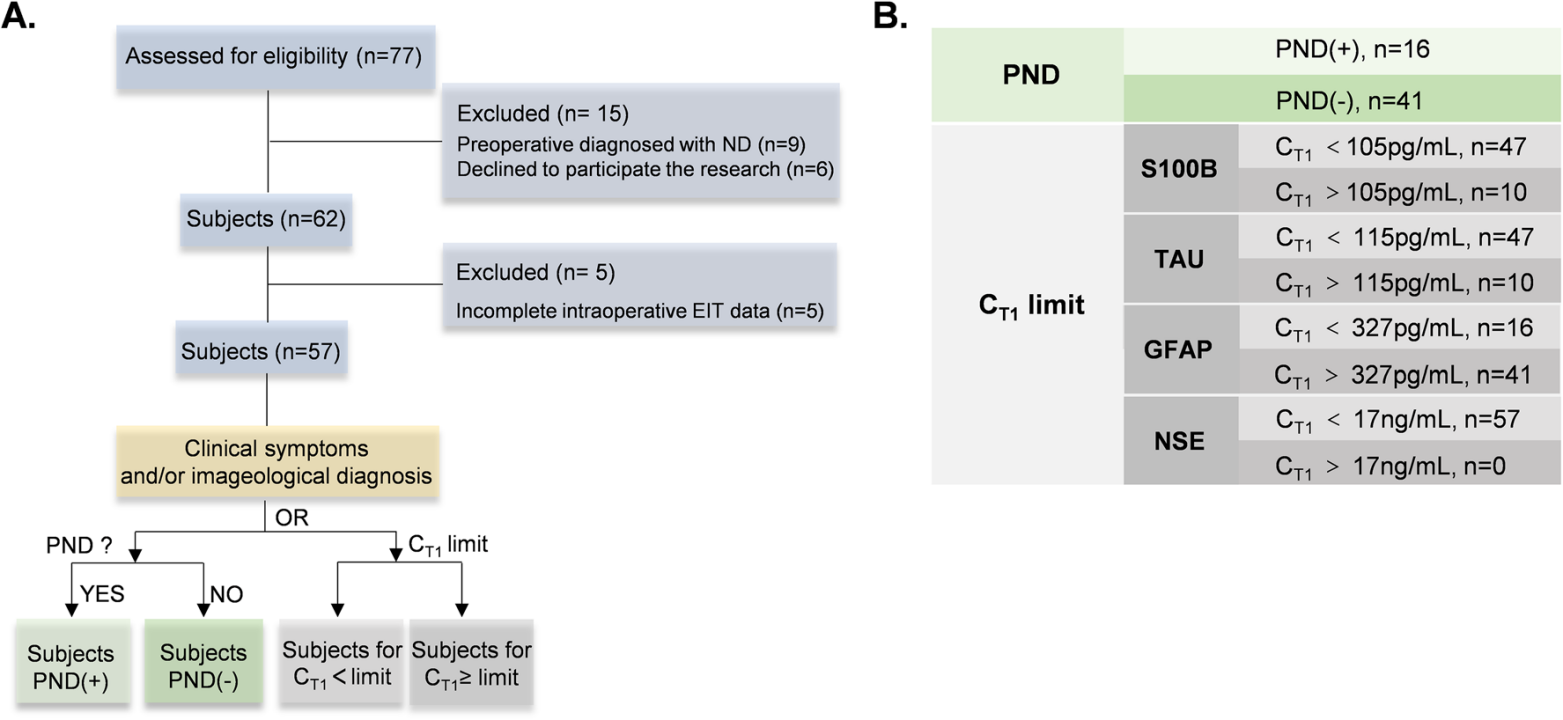
Enrollment of study population and classification method for data analysis. (**A**) The flowchart illustrating the enrollment of study population. (**B**) The classification of data for analyzation. *ND* neurological dysfunction, *PND* postoperative neurological dysfunction, *PND (*+*)* patients with PND, *PND (−)* patients without PND, *T1* time before surgery, *C*_*T1*_ concentration of neurological biomarkers in serum at time T1.

From December 2021 to March 2022, 77 patients with aortic dissection type A admitted at Xijing Hospital of Fourth Military Medical University were included in this study. There were 9 out of 77 were excluded because of preoperative neurological dysfunction and other 6 patients declined to participate. In addition, 5 subjects were excluded because their incomplete EIT data due to inappropriate operation during EIT data collection. Finally, data from 57 subjects were analyzed, among which 16 subjects were defined as PND, through clinical examinations, and 41 subjects were classified in non PND group (Fig. 1A). The classification method for data analysis was shown in Fig. 1B.

The characteristics of total patients were summarized in Table 1. The average age of total patients (male 48, 84.2%) was 50.5 (8.5) years old. Only 1 patient was with Marfan syndrome, 2 patients had diabetes and 2 had history of coronary heart disease. Out of 57 patients, 37 had hypertension and 34 patients had smoking history. In addition, 53 patients had acute aortic dissection, 4 patients had syncope, 16 out of 57 had aortic valve insufficiency, 17 patients had intramural hematoma and 28 had aortic arch branches involvement. The average operation time was 386.8 ± 54.5 min, the average CPB time was 219.3 (26.4) min and the average HCA time was 36.1 (9.5) min. Moreover, the average days for ICU stay were 8.3 (6.5) days for PND (+) and 5.4 (4.6) days for PND (−). The in-hospital death happened to 2 patients with PND (+). Other information in detail, including demographics and comorbidities, preoperative data, procedural data and ICU data and mortality, were listed in Table 1.

**Table 1:** Characteristics of patients with and without postoperative neurological dysfunction (PND (+) and PND (−)).

Variables	All (N = 57)	PND ( +) (N = 16)	PND ( −) (N = 41)	*p*-value
Demographics and comorbidities				
Gender, male	48 (84.2%)	14(87.5%)	34 (82.9%)	0.670
Age (year)	50.5 (8.5)	49.5 (8.6)	50.9 (8.5)	0.590
BMI (kg/m^2^)	24.9 (3.8)	26.4 (4.1)	24.3 (3.5)	0.085
Coronary heart disease	2 (3.5%)	1 (6.3%)	1 (2.4%)	0.486
Marfan syndrome	1 (1.7%)	1 (6.3%)	0 (0%)	0.435
Hypertension	37 (64.9%)	11 (68.8%)	26 (63.4%)	0.704
Diabetes	2 (3.5%)	1 (6.3%)	1 (2.4%)	0.486
Chronic kidney disease	4 (7%)	1 (6.3%)	3 (7.3%)	1.000
Smoking history	34 (59.6%)	11 (68.8%)	23 (56.1%)	0.382
History of open-heart surgery	1 (1.8%)	0 (0%)	1 (2.4%)	1.000
Preoperative data				
Acute aortic dissection	53 (93.0%)	16 (100%)	37 (90.2%)	0.568
Syncope	4 (7.0%)	2 (12.5%)	2 (4.9%)	0.312
Lower limb ischemic syndrome	12 (21.1%)	3 (18.8%)	9 (22.0%)	0.790
NYHA III/IV	23 (40.3%)	9 (56.3%)	14 (34.1%)	0.378
LVEF (%)	48.8 (6.1)	49.7 (8.2)	48.4 (5.1)	0.574
Aortic valve insufficiency	16 (28.1%)	7 (43.8%)	9 (22.0%)	0.100
Intramural hematoma	2 (3.5%)	1 (6.3%)	1 (2.4%)	0.486
Pericardial effusion	17 (29.8%)	4 (25.0%)	13 (31.7%)	0.619
Aortic arch branches involvement	28 (66.7%)	7 (53.8%)	21 (72.4%)	0.238
Serum creatinine (μmol/L)	107.9 (53.9)	134.1 (50.8)	97.1 (52.0)	0.021
Procedural data				
Combined with Bentall procedure	25 (43.9%)	10 (62.5%)	15 (36.6%)	0.076
Combined with CABG procedure	4 (7.0%)	2 (12.5%)	2 (4.9%)	0.312
Double arterial cannulation	34 (59.6%)	9 (56.3%)	25 (61.0%)	0.744
Innominate artery cannulation	23 (40.4%)	6 (37.5%)	17 (41.5%)	0.784
Axillary artery cannulation	25 (43.9%)	6 (37.5%)	19 (46.3%)	0.546
Femoral artery cannulation	41 (71.9%)	12 (75.0%)	29 (70.7%)	0.747
Bilateral ACP	33 (57.9%)	7 (43.8%)	26 (63.4%)	0.177
Operation time (min)	386.8 (54.5)	404.3 (54.9)	380.2 (53.6)	0.173
CPB time (min)	219.3 (26.4)	230.3 (31.0)	215.1 (23.4)	0.091
Aortic clamp time (min)	105.5 (17.0)	110.1 (19.3)	103.6 (15.9)	0.244
HCA time (min)	36.1 (9.5)	35.4 (6.2)	36.4 (10.6)	0.663
Hypothermia temperature (°C)	25.4 (0.6)	25.3 (0.8)	25.4 (0.5)	0.586
ICU data and mortality				
Delayed awakening (> 6 h)	5 (8.8%)	0 (0%)	5 (12.2%)	0.308
Ventilation > 24 h	25 (41.7%)	8 (50.0%)	16 (39.0%)	0.413
ICU stay (days)	6.2 (5.3)	8.3 (6.5)	5.4 (4.6)	0.121
Hemodialysis	4 (7.0%)	3 (18.8%)	1 (2.4%)	0.063
In-hospital death	2 (3.5%)	2 (12.5%)	0 (0%)	0.075

Data are presented as mean (SD) or n (%).

*BMI* body mass index, *NYHA* New York Heart Association, *LVEF* left ventricular ejection fraction, *ACP* antegrade cerebral perfusion, *CPB* cardiopulmonary bypass, *HCA* hypothermic circulatory arrest, *ICU* intensive care unit.

### Classification of EIT extracted parameters based on PND

**Figure 2:**
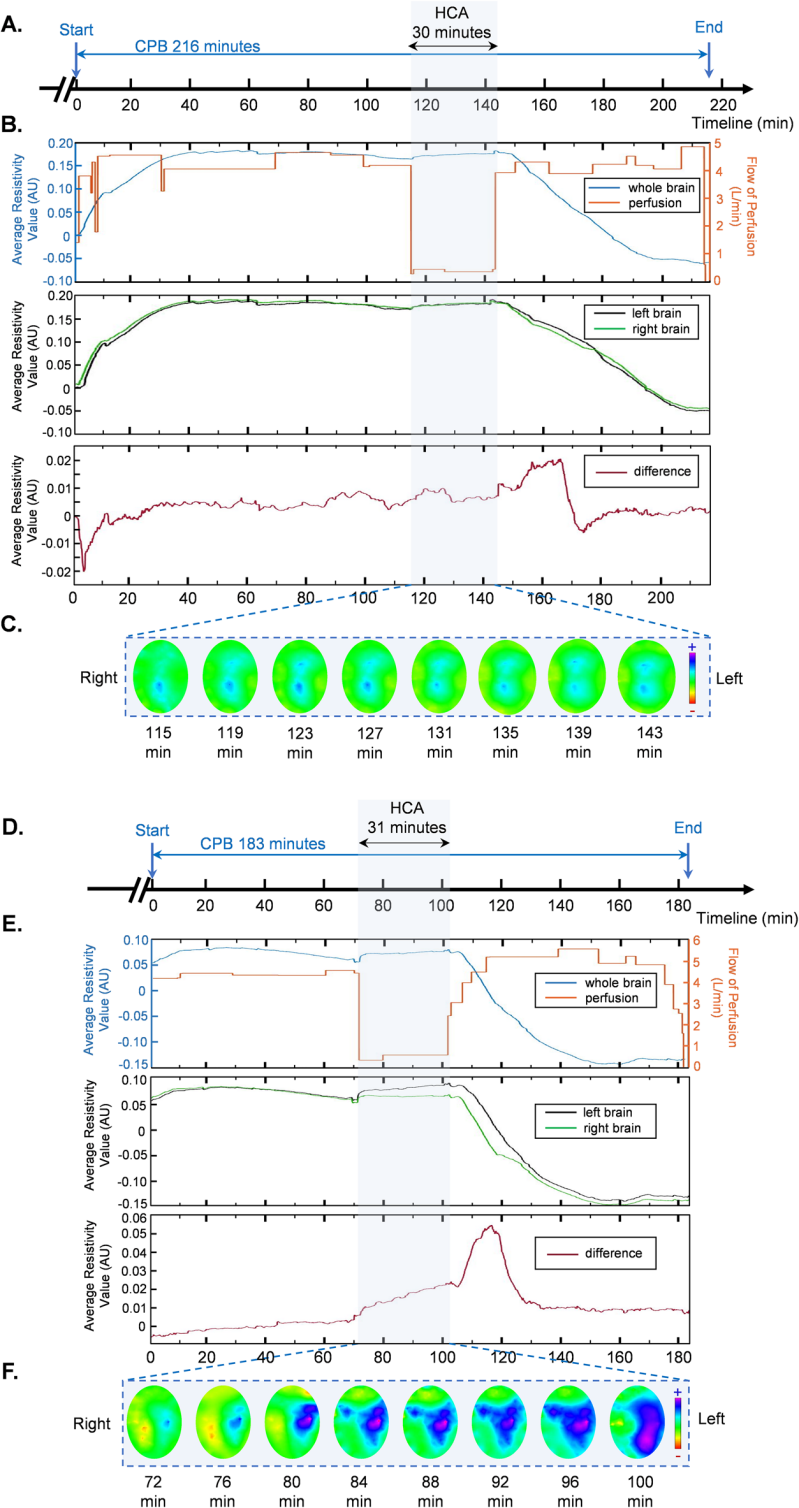
Timeline of CPB in the surgery and representative cerebral electrical impedance tomography during HCA. (**A,D**) Timeline of monitoring and CPB period for patients PND (−) and PND (+) respectively. (**B,E**) The representative EIT data of the whole brain and the flow of perfusion (top panel), EIT data of left brain and right brain (middle panel) and difference of electrical impedance between left and right brain (bottom panel) for PND (−) and PND (+) respectively. (**C,F**) Representative cerebral EIT pictures during HCA phase. *CPB* cardiopulmonary bypass, *HCA* hypothermic circulatory arrest phase, *EIT* electrical impedance tomography, *PND (*+*)* patients with postoperative neurological dysfunction, *PND (−)* patients without postoperative neurological dysfunction.

Cerebral EIT signals were monitored and recorded during whole CPB period (Fig. 2A,D). The overall trend of EIT signal for patient with PND was different from that without PND (Fig. 2B,E). Representative cerebral electrical impedance information for the two groups were showed in Fig. 2. The green color line described the average resistivity value of right brain during operation and the black line displayed the average resistivity value of left brain. For the patients without PND, there was little difference between left and right brain (Fig. 2B), while for the patient with PND, the difference value between two hemispheres of the brain was significant (Fig. 2E). In addition, the electrical impedance tomography was displayed during HCA phase for each 30 min (Fig. 2C,F).

EIT parameters were extracted and calculated according to Eqs. (1)–(7). None of the data conformed to normal distribution. The correlation coefficients among five parameters were higher in patients with PND than those in patients without PND (Supplementary file: Tables S1–S3). The maximum value of the absolute difference of electrical impedance between left and right brain (MRAI_abs_) was significantly higher in patients with PND than that in patients without PND (0.46 (0.28–0.85) vs. 0.29 (0.14–0.49), *p* = 0.044), and absolute value of the time integral of RAI (TRAI_abs_) was also significantly higher in PND(+) compared to that in PND(−) (732.74 (348.47–1710.19) vs. 344.71 (121.12–789.06), *p* = 0.020) (Table 2). However, after applying multiple testing correction, there was no significant difference between groups.

**Table 2:** EIT extracted parameters for patients with and without postoperative neurological dysfunction (PND (+) and PND (−)).

EIT parameters	PND (+) N = 16	PND (−) N = 41	*p* value
ΔARV_HCA_	0.03 (− 0.05 to 0.16)	0.08 (− 0.06 to 0.44)	0.657
k_HCA_ (× 10^5^)	2.01 (− 1.82 to 6.83)	4.3 (− 2.74 to 17.96)	0.607
MRAI_abs_	0.46 (0.28 to 0.85)	0.29 (0.14 to 0.49)	0.044*
TRAI_abs_	732.74 (348.47 to 1710.19)	344.71 (121.12 to 789.06)	0.020*
TARV_HP_	− 0.83 (− 14.3 to 42.04)	− 12.83 (1.84 to 29.64)	0.804

Data are presented as median (IQR).

*HCA* hypothermic circulatory arrest, *ΔARV*_*HCA*_ the difference of average resistivity value before and after HCA phase, *k*_*HCA*_ the slope of electrical impedance during HCA phase, *MRAI*_*abs*_ maximum of the absolute value of resistivity asymmetric index, *TARI*_*abs*_ absolute value of time integral of resistivity asymmetric index, *TARV*_*HP*_ time integral of electrical impedance for half flow of perfusion.

*p value < 0.05 means the value was significantly different between PND(+) and PND(−).

**Figure 3:**
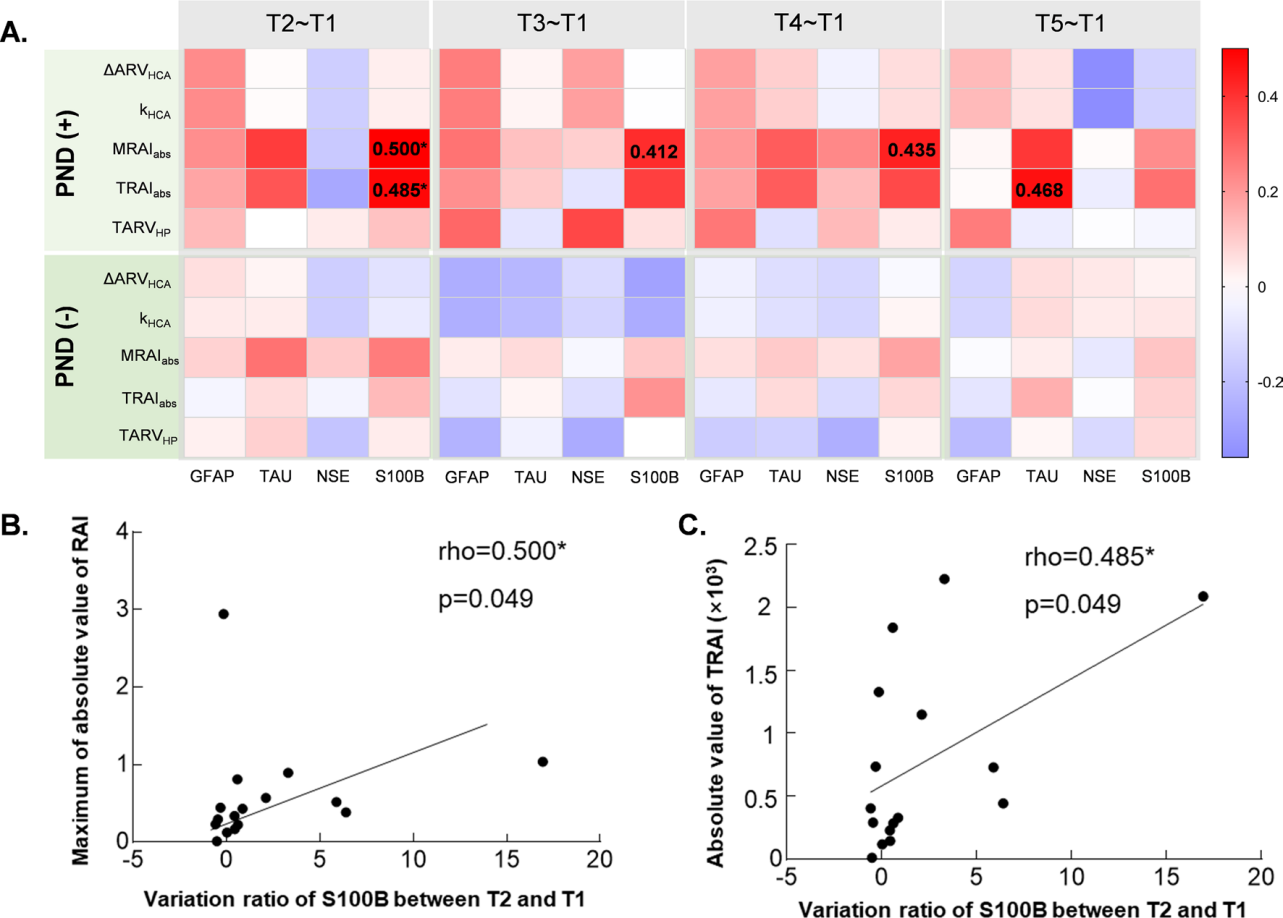
Correlation between relative difference of serum biomarkers and EIT extracted parameters for patients with and without PND. (**A**) The heatmap of correlation coefficient and significant correlations can be seen in PND (+) group while no significant correlation in PND (−) group. (**B**) The correlation of MRAI_abs_ and relative difference of S100B protein between T2 and T1 for patients with PND. (**C**) The correlation of TARI_abs_ and relative difference of S100B protein between T2 and T1 for patients with PND. *PND (−)* patients without postoperative neurological dysfunction, *PND (*+*)* patients with postoperative neurological dysfunction, *T2–T1* relative difference between after and before surgery, *T3–T1* relative difference between 12 h after and before surgery, *T4–T1* relative difference between 24 h after and before surgery, *T5–T1* relative difference between 48 h after and before surgery, *HCA* hypothermic circulatory arrest, *ΔARV*_*HCA*_ the difference of average resistivity value before and after HCA phase, *k*_*HCA*_ the slope of electrical impedance during HCA phase, *MRAI*_*abs*_ maximum of the absolute value of resistivity asymmetric index, *TARI*_*abs*_ absolute value of time integral of resistivity asymmetric index, *TARV*_*HP*_ time integral of electrical impedance for half flow of perfusion; *means p < 0.05.

The correlation coefficient between relative difference of four serum biomarkers and five EIT parameters were displayed in a heatmap (Fig. 3A). Coefficients in detail were listed in Supplementary file: Table S9. The upper half part was for patients with PND and the lower half part was for those without PND. It appears that the correlation coefficients were generally higher in the upper part than that in the lower part, which revealed that neurological biomarkers correlated with EIT extracted parameters more in patients with PND than that in patients without PND. Particularly, the coefficients for MRAI_abs_ and TRAI_abs_ with the relative difference of S100B-T2T1 were 0.500 and 0.485 respectively, indicating that there was significant moderate correlation between relative difference of S100B 48 h postoperative respect to preoperative level for MRAI_abs_ and TRAI_abs_ (Fig. 3B,C).

### Classification of data based on the level of biomarkers in serum at T1

Due to the individual difference in the involvement degree of dissection in aortic arch branches, some patients developed mild brain injuries before surgery, which led to the inconsistence of preoperative biomarker levels^24,25^. Therefore, we grouped patients according to the normal threshold of serum biomarkers at T1, then the correlation between EIT parameters and relative differences of serum biomarkers for each group.

**Figure 4:**
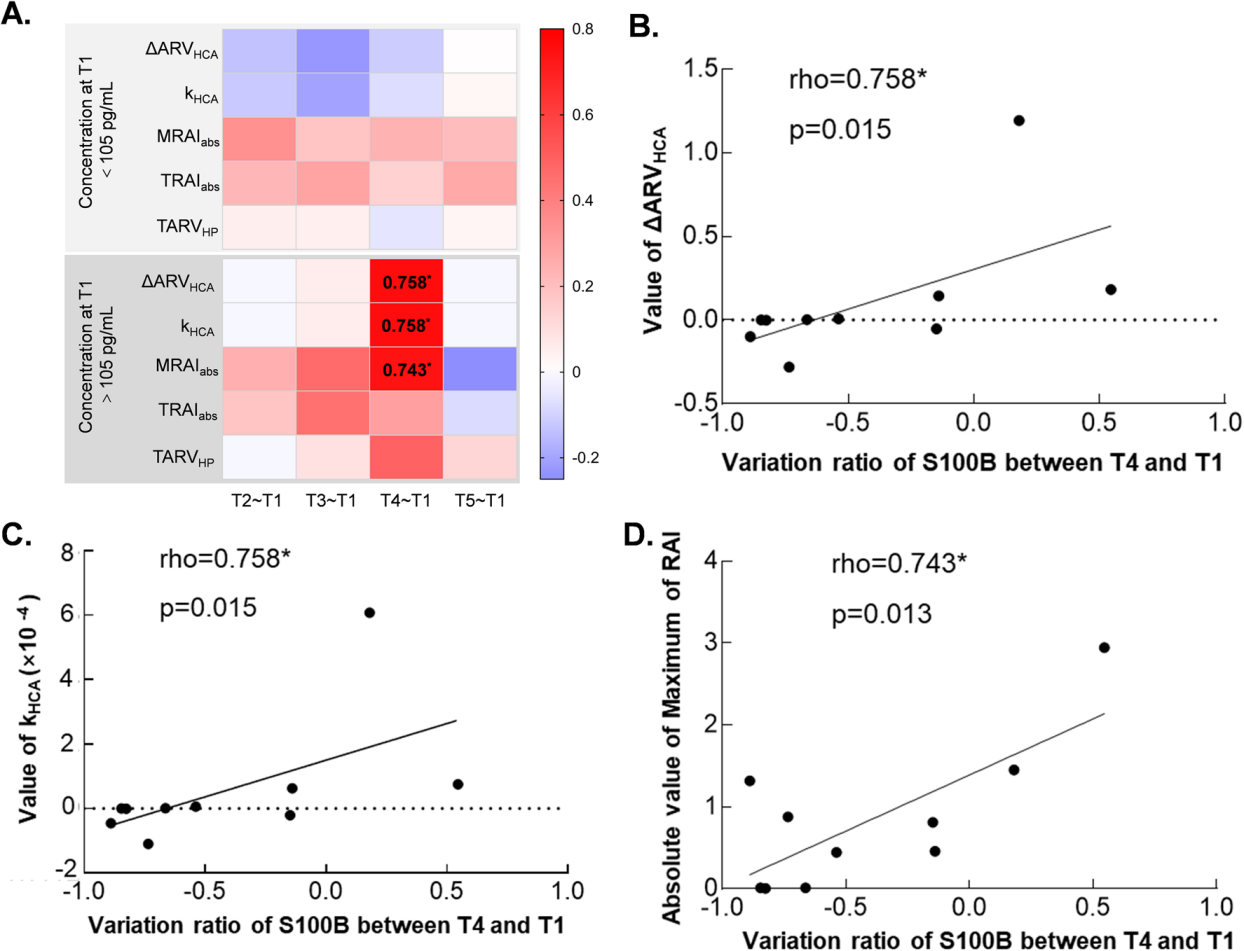
Correlation between relative difference of serum biomarkers and EIT extracted parameters for patients with normal and abnormal concentration of S100B before surgery. (**A**) The heatmap of correlation coefficient between relative difference of S100B protein and EIT extracted information. Significant correlations can be seen in group of C_T1_-S100B > 105 pg/mL. (**B**) Correlation of ΔARV_HCA_ and relative difference of S100B protein between T4 and T1 for patients with C_T1_-S100B > 105 pg/mL. (**C**) The correlation of k_HCA_ and relative difference of S100B protein between T4 and T1 for patients with C_T1_-S100B > 105 pg/mL. (**D**) The correlation of MRAI_abs_ and relative difference of S100B protein between T4 and T1 for patients with C_T1_-S100B > 105 pg/mL. *T2–T1* relative difference between after and before surgery, *T3–T1* relative difference between 12 h after and before surgery, *T4–T1* relative difference between 24 h after and before surgery, *T5–T1* relative difference between 48 h after and before surgery, *HCA* hypothermic circulatory arrest, *ΔARV*_*HCA*_ the difference of average resistivity value before and after HCA phase, *k*_*HCA*_ the slope of electrical impedance during HCA phase, *MRAI*_*abs*_ maximum of the absolute value of resistivity asymmetric index, *TARI*_*abs*_ absolute value of time integral of resistivity asymmetric index, *TARV*_*HP*_ time integral of electrical impedance for half flow of perfusion; *means p < 0.05.

For S100B protein, the level of concentration in serum of healthy human is around 105 pg/mL^26^. Coefficients in detail were listed in Supplementary file: Table S10. In general, we found that the correlation coefficients were positively greater in the patients with a S100B level higher than 105 pg/mL (Fig. 4A). In particularly, the variation of S100B after 24 h were significantly strong correlated with ΔARV_HCA_, k_HCA_ and MRAI_abs_ for patients with serum S100B level greater than 105 pg/mL. The Spearman coefficient were 0.758 (*p* = 0.015), 0.758 (*p* = 0.015) and 0.743 (p = 0.013) for correlation between S100B and ΔARV_HCA_, k_HCA_ and MRAI_abs_, respectively (Fig. 4B–D).

**Figure 5:**
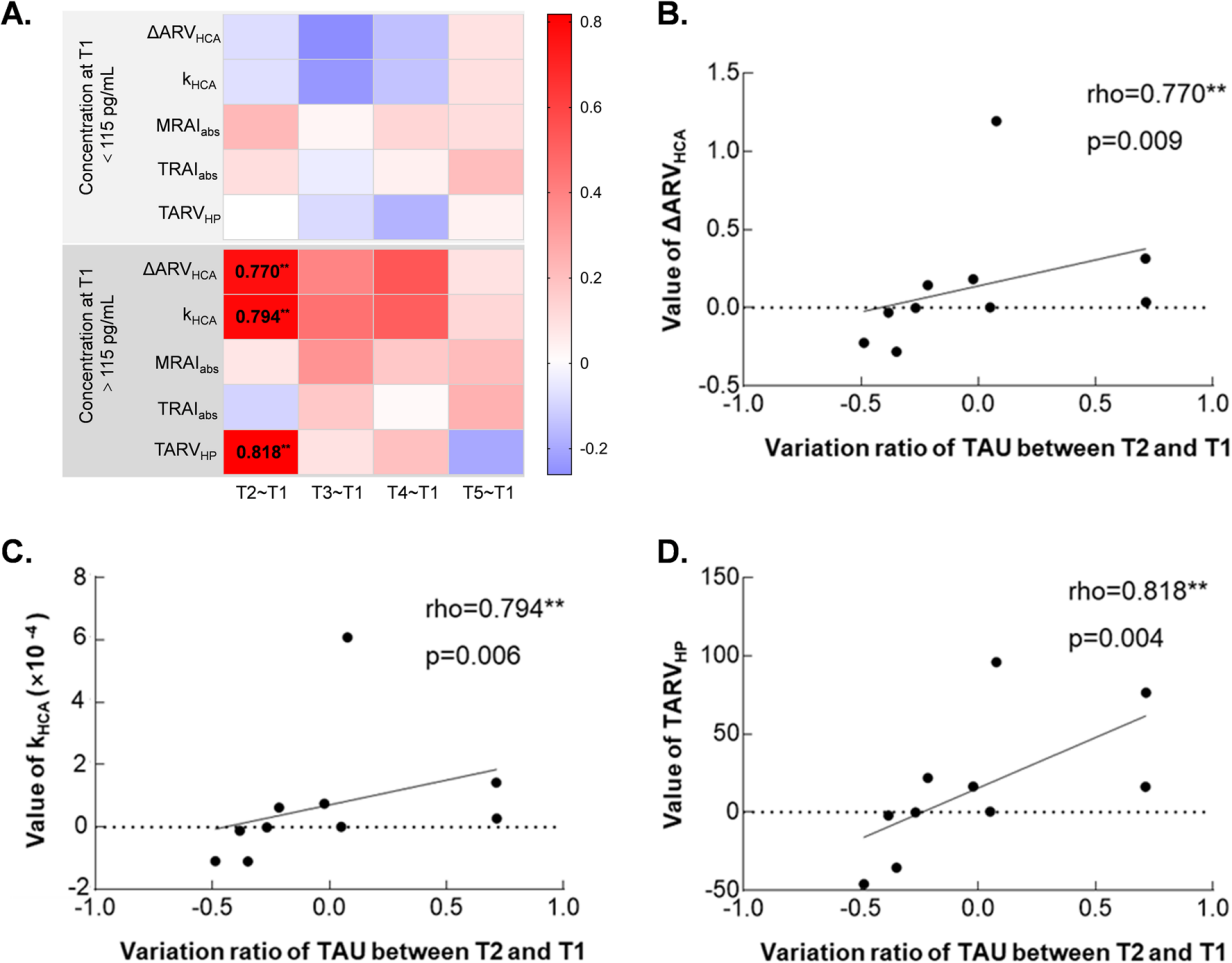
Correlation between relative difference of serum biomarkers and EIT extracted parameters for patients with normal and abnormal concentration of TAU before surgery. (**A**) Heatmap of correlation coefficient between relative difference of TAU protein and EIT extracted information. Significant correlations can be seen in group of C_T1_-TAU > 115 pg/mL. (**B**) The correlation of ΔARV_HCA_ and relative difference of TAU protein between T2 and T1 for patients with C_T1_-TAU > 115 pg/mL. (**C**) The correlation of k_HCA_ and relative difference of TAU protein between T2 and T1 for patients with C_T1_-TAU > 115 pg/mL. (**D**) The correlation of TARV_HP_ and relative difference of TAU protein between T2 and T1 for patients with C_T1_-S100B > 115 pg/mL. *T2–T1* relative difference between after and before surgery, *T3–T1* relative difference between 12 h after and before surgery, *T4–T1* relative difference between 24 h after and before surgery, *T5–T1* relative difference between 48 h after and before surgery, *HCA* hypothermic circulatory arrest, *ΔARV*_*HCA*_ the difference of average resistivity value before and after HCA phase, *k*_*HCA*_ the slope of electrical impedance during HCA phase, *MRAI*_*abs*_ maximum of the absolute value of resistivity asymmetric index, *TARI*_*abs*_ absolute value of time integral of resistivity asymmetric index, *TARV*_*HP*_ time integral of electrical impedance for half flow of perfusion; **means p < 0.01.

As for TAU protein, the limit was set on 115 pg/mL according to our laboratory results for normal people (data not shown). Interestingly, the correlation of EIT extracted parameters had greater correlation with relative difference of TAU protein for patients with an abnormal level than those with normal level before surgery (Fig. 5A). Coefficients in detail were listed in Supplementary file: Table S11. In particularly, the relative difference of TAU right after surgery had a positively strong correlation with ΔARV_HCA_, k_HCA_ and TARV_HP_ (0.770 with *p* = 0.009, 0.794 with 0.006 and *p* = 0.818 with *p* = 0.004, respectively) (Fig. 5B–D).

**Figure 6:**
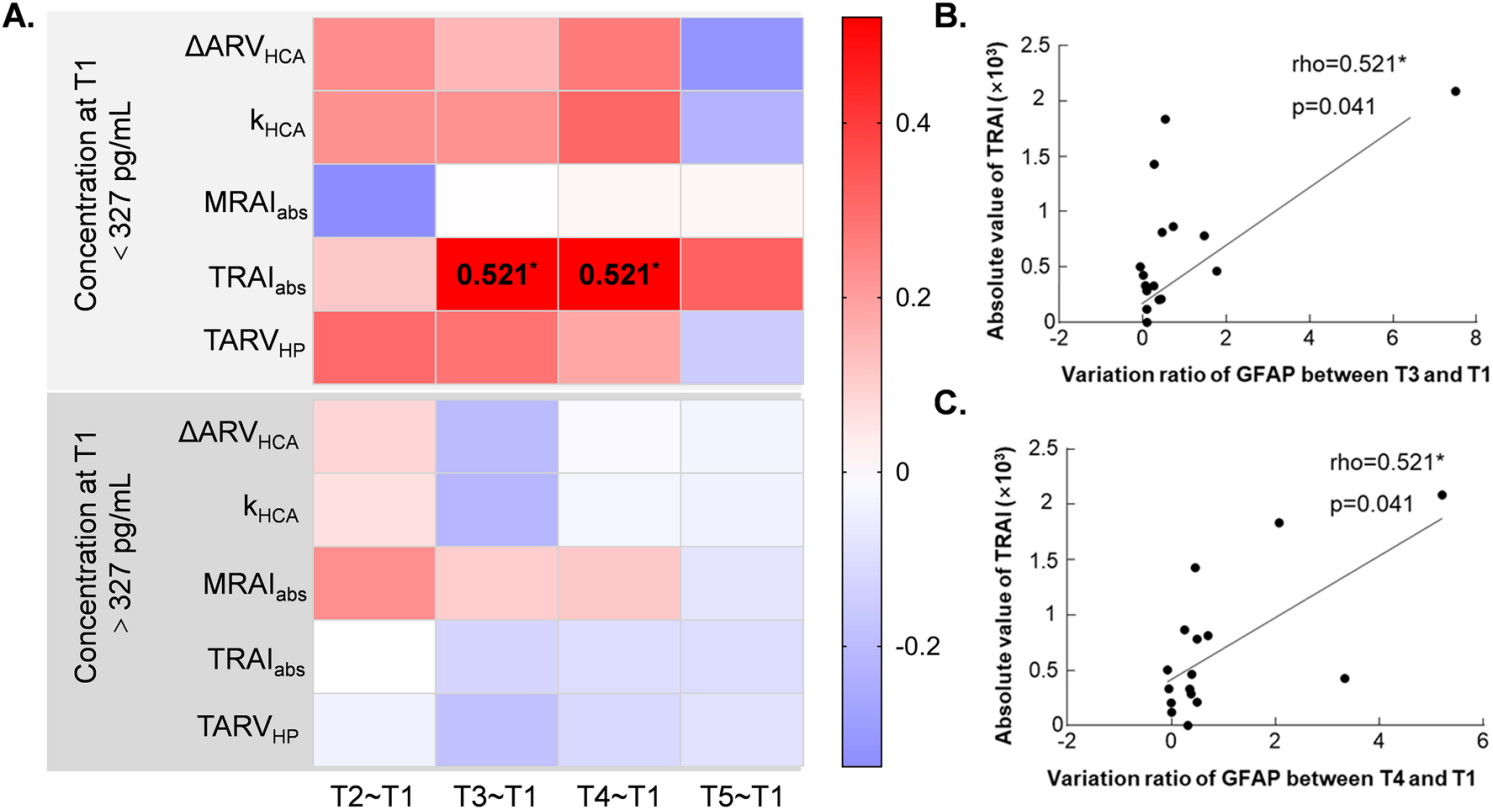
Correlation between relative difference of serum biomarkers and EIT extracted parameters for patients with normal and abnormal concentration of GFAP before surgery. (**A**) Heatmap of correlation coefficient between relative difference of GFAP protein and EIT extracted information. Significant correlations can be seen in group of C_T1_-GFAP < 327 pg/mL. (**B**) The correlation of TARI_abs_ and relative difference of GFAP protein between T3 and T1 for patients with C_T1_-GFAP < 327 pg/mL. (**C**) The correlation of TARI_abs_ and relative difference of GFAP protein between T4 and T1 for patients with C_T1_-GFAP < 327 pg/mL. *T2–T1* relative difference between after and before surgery, *T3–T1* relative difference between 12 h after and before surgery, *T4–T1* relative difference between 24 h after and before surgery, *T5–T1* relative difference between 48 h after and before surgery, *HCA* hypothermic circulatory arrest, *ΔARV*_*HCA*_ the difference of average resistivity value before and after HCA phase, *k*_*HCA*_ the slope of electrical impedance during HCA phase, *MRAI*_*abs*_ maximum of the absolute value of resistivity asymmetric index, *TARI*_*abs*_ absolute value of time integral of resistivity asymmetric index; *TARV*_*HP*_ time integral of electrical impedance for half flow of perfusion; *means p < 0.05.

**Figure 7:**
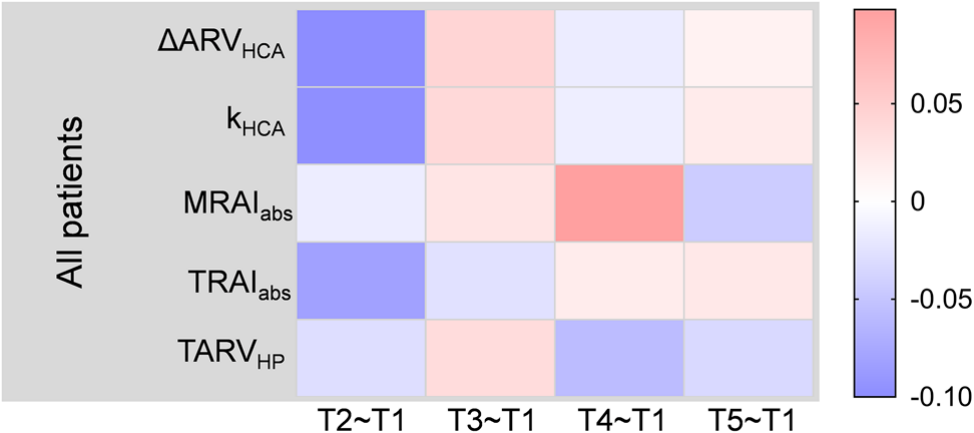
Heatmap of correlation coefficient between relative difference of NSE protein and EIT extracted information for all patients. No significant correlations can be seen. *T2–T1* relative difference between after and before surgery, *T3–T1* relative difference between 12 h after and before surgery, *T4–T1* relative difference between 24 h after and before surgery, *T5–T1* relative difference between 48 h after and before surgery, *HCA* hypothermic circulatory arrest, *ΔARV*_*HCA*_ the difference of average resistivity value before and after HCA phase, *k*_*HCA*_ the slope of electrical impedance during HCA phase, *MRAI*_*abs*_ maximum of the absolute value of resistivity asymmetric index, *TARI*_*abs*_ absolute value of time integral of resistivity asymmetric index, *TARV*_*HP*_ time integral of electrical impedance for half flow of perfusion.

On the contrary, the correlation of EIT extracted parameters had greater correlation with relative difference of GFAP protein for patients with normal level than those with abnormal level before surgery (Fig. 6A). Coefficients in detail were listed in Supplementary file: Table S12. Significant moderate correlation can be seen in the variation of 12 h and 24 h after surgery with TRAI_abs_, which was 0.521 (*p* = 0.041) and 0.521 (*p* = 0.041) respectively (Fig. 6B,C). However, all NSE level at T1 were below the limit, which was 17 ng/mL^26^, and none of them had significant correlation with any of those EIT parameters (Fig. 7). Coefficients in detail were listed in Supplementary file: Table S13.

## Discussion

In this study, we obtained five specific parameters that extracted from EIT monitored during HCA period and analyzed the relationship between EIT parameters and four neurological biomarkers in serum in order to explore the potential of EIT parameters as early indicators of PND for patients underwent TAAR. Of the five parameters and biomarkers, two parameters represented for the difference of electrical impedance between left and right brain (TRAI_abs_ and MRAI_abs_) had the strongest correlation with neurological biomarkers indicating that these two parameters had a great potential to correspond hypoxic-ischemic brain damage with physiological status in real time and they could be the independent predictors of PND for patients underwent TAAR.

As we know, during TAAR operation, the brain is at risk of ischemia due to the interruption of blood flow^1^. Therefore, it is critical to monitor the brain for any signs of ischemia or other neurologic issues that may arise^5^. Current noninvasive intraoperative devices for brain monitoring during surgery includes those for recording neurophysiological functions, techniques that assess hemodynamic changes and instruments that measure cerebral oxygenation^5,24^. The descriptions of different devices along with their pros and cons were listed in Table 3. EIT is a non-invasive, inexpensive and portable imaging technique that involves placing electrodes on the scalp and measuring changes of electrical impedance within the brain tissue in real time^27^. Similar to EEG, EIT is one of the devices recording cerebral electrical properties through collecting and analyzing potentials. Compared to EEG, or the quantitative EEG, EIT allow us to obtain the information not only focused on the superficial layers of cortex but importantly the global brain (Table 3). However, it is noted that none of these methods are foolproof and may not detect all neurological deficits^24^. A multimodal approach is recommended for accurate monitoring of cerebral injury during TAAR surgery^28^. Our group firstly applied EIT in cerebral imaging and monitoring in patients underwent TAAR, proving that EIT had the potential to provide quantitative information of regional cerebral perfusion^14,16^.

**Table 3:** Overview of advantages and disadvantages for intraoperative noninvasive neuromonitoring techniques.

Techniques	Description	Advantages	Disadvantages
EEG^24^; Quantitative EEG^42^ (e.g., BIS and PSI)	An EEG records the spontaneous electrical activity in the brain via electrodes attached to the scalp Quantitative EEG applies mathematical approaches to obtain a quantitative description of EEG	Easy to apply and interpret Be able to record the electrical inactivity in brain during anesthesia	Measurements focused on superficial layers of cortex One-dimensional signals Delay in processing for quantitative EEG
Somatosensory evoked potentials^43^	Determination of the integrity of neural pathways by stimulation and recording of known electric signals	Quantitative information Detection of cerebral metabolic suppression	Susceptible to interference (e.g., anesthesia) Delay in interpretation
EIT^14,16,44^	EIT estimates the electrical properties at the interior of brain based on reconstruction of resistivity of different tissues	Real-time and continuous Functional and visualization High sensibility to pathophysiological changes in brain	Novel Susceptible to movements More cross validation with other techniques needed
NIRS^45^	NIRS monitors regional tissue oxygenation by different absorption wavelengths of near-infrared light of oxyhemoglobin and deoxy hemoglobin	Easy to apply Low cost	Limited in frontal region Not well-validated on ability to detect ischemia or cognitive dysfunction Delay in processing
TCD^46^	TCD records the speed of the blood flow in arteries known as the Circle of Willis using a 2 MHz focused pulsed Doppler ultrasound	Direct visualization of blood flow distribution Live feedback Detection of cerebral microemboli	Specialized training and expertise required Limited by acoustic
LSFG of ophthalmic artery^47^	Recording the blood flow by speckle patterns caused from laser scattered by red blood cells	Quantitative evaluation Real-time	Not well validated

*EEG* electroencephalogram, *BIS* bispectral index, *PSI* patient state index, *EIT* electrical impedance tomography, *NIRS* near-infrared spectroscopy, *TCD* transcranial Doppler, *LSFG* laser speckle flowgraphy.

During TAAR surgery, a period of arrest of CPB is necessary for completion of cardiovascular surgery involving heart and great vessels. Hypothermia circulation arrest was meant to be protective for brain and other important organs from ischemic injury^29^. However, previous research showed that cerebral injury could appear in HCA phase due to the sudden change of oxygen supply to the brain^1^. Therefore, in this study we focused on the EIT information in HCA phase. In order to build a sound relationship between EIT information and the degree of brain damage during operation which could lead to neurological dysfunction after surgery, we extracted five parameters based on EIT signals around HCA period. We found that the difference of electrical impedance between left and right brain were significantly different between PND (+) and PND (−) (Table 2). However, although the difference was significant from the view of EIT signals (Fig. 2) and significant differences exists in our previous study^16^, *p*-values tended to be slightly greater than 0.05 after a multiple testing correction in this research. We supposed that the instability of results was due to the small size of samples, especially those for PND (+). Moreover, PND is a series of clinical symptoms which are results from physiological and pathological changes during whole perioperative period, in order to clarify the practicability of EIT from a more objective and statistical point of view, investigation of correlation between these parameters and physiological indicators was necessary.

Interestingly, neurological biomarkers, including S100B, TAU, GFAP and NSE protein, can be detected with a significant variation in serum when neurological dysfunction occurs^18^. S100B is a calcium-binding protein that is predominantly expressed in astrocytes and can be used as a biomarker for acute brain injury^22^. In a randomized clinical trial, the results showed that for patients with brain injury, the serum S100B increased during CPB with a peak plasma concentration at the termination of CPB and gradually decreased after 24 hours^30^. In our study, compared to patients without PND, PND (+) patients had an increasing of S100B serum level after TAAR with a peak concentration right after surgery and returned to normal level 24–48 h after surgery, which was consistent with previous studies (Tables S7, S8)^30,31^. TAU protein is a microtuble-associated protein found in neurons and is a biomarker for neurodegeneration^32,33^. In an observational study of long-term persistent elevation of neurodegeneration markers after cardiac surgery, researchers found that serum levels of TAU protein increased 24 h after surgery and remained elevated at 7 days and three months for patients with neurodegeneration^34^. Although we did not test the serum level in a long term, we did find that the TAU level increased after surgery and remained elevated at 48 h for patients with PND (Tables S7, S8).

In addition, GFAP is a protein that found in astrocytes and is released into the bloodstream following brain injury, and it has a high specificity and sensitivity of neurological damages^32^. Nurcahyo et al. examined GFAP serum levels for patients with and without postoperative cognitive dysfunction (POCD) after on-pump coronary artery bypass grafting^35^. Their study showed that POCD patients had higher GFAP levels than non-POCD patients, which was consistent with our results in ELISA tests of GFAP levels in serum (Tables S7, S8). Finally, NSE is an enzyme found in neurons and neuroendocrine cell and can be adopted as a marker of neurological damage^36^. Some researchers indicated that the released NSE are associated with patients’ clinical deficits and infarct volume while there was no significant difference between patients with PND (+) and PND (−) in another study^23,37^. Our study showed that the serum level of NSE was generally higher in PND (+) than that in PND (−) yet without significance.

Given that there were differences in both increase time and time to peak value for four biomarkers’ concentration, we collected the blood samples at five time points which were before surgery, right after surgery, 12 h, 24 h and 48 h after surgery, so that the peak value for each biomarker concentration can be included. The relationship was exploited between each EIT parameters and the relative difference of each serum biomarker for each time interval. Firstly, total subjects were divided into two groups, PND (+) and PND (−), depending on their clinical syndromes and image test results. From the heatmap, we can tell that the correlation between EIT parameters and serum biomarkers were stronger in PND (+) than that in PND (−) on the whole (Fig. 3A). Specifically, relative difference of S100B protein right after surgery was significantly correspondent with two EIT parameters (Fig. 3B,C), which seemed to be explainable with previous studies on correlation of serum biomarkers and neurological dysfunction, in which around 90% of specificity and sensitivity was found in S100B level after a cerebral injury after a surgery with HCA^19^. These results indicated that the asymmetric electrical impedance between left and right brain during HCA period might be independent predictors for brain damage.

Afterwards, we classified patients based on whether the serum biomarker level before surgery was below the normal value based on our laboratories reference standards or from literature^18,19,23,26,38^. Results showed that for patients with an abnormal serum level of S100B, the correlation between its variation 24 h after surgery and three EIT extracted parameters, namely ΔARV_HCA_, k_HCA_ and MRAI_abs_, were strong and significant (Fig. 4). Since serum level S100B arrived at peak value around 24 h after cardiac surgery^30,31^ (Table S7), we believed that these three EIT parameters along with S100B had great potential for giving information of neurological damage during surgery for patients with an abnormal preoperative neurological function. Additionally, EIT parameters ΔARV_HCA_, k_HCA_ and TARV_HP_ had great correlation with variation of serum TAU concentration right after surgery for patients with an abnormal serum level before surgery (Fig. 5). Actually, serum TAU level reached a peak value right after surgery and there was a significant difference between PND (+) and PND (−) in our study (Table S7), which could explain the great correlation with its variation and EIT parameters.

For the biomarker GFAP, significant correlation can be found in patients with a normal serum level before surgery, and the EIT extracted parameter TRAI_abs_ had good correlation with both variation of GFAP 12 h and 24 h after surgery (Fig. 6). Researchers reviewed the evidence regarding the utility of serum GFAP as a biomarker in neurological diseases and they believed that clinical use of GFAP measurements had the potential to contribute to accelerated diagnosis and improved prognostication^38^. Finally, no significant correlation was found in variation of NSE and any of EIT extracted parameters (Fig. 7). Actually, studies showed that no significant difference of serum NSE was found between patients with and without neurological dysfunction^23^. The reason may be that NSE was released not only by neurons but it also existed in blood platelet and red blood cells^39^.

Although the correlation between EIT parameters and variation of serum biomarkers in the PND (+) group was higher than that in the PND (−) group as a whole, it did not show a strong linear correlation between any of those EIT parameters and biomarkers. First, some data showed that the concentration of biomarkers in serum at T1 were beyond the normal range, indicating that these patients had a certain degree of brain injury before surgery. These brain lesions are so small that there were neither clinical symptoms nor imaging indications, yet confounding the correlation analyses. Second, we studied the EIT parameters during HCA phase, however, brain injury could be induced by several factors in the whole process of CPB period, especially in the rewarming phase^1,2^. Actually, we are exploring and analyzing EIT parameters from the perspective of whole process of surgery for the future research.

Some limitations in this study should be clarified. First of all, although operators in clinical studies were professionally trained and had at least 5 years of work experience, there was still an inevitable problem of consistency due to the individual differences of patients. Afterwards, a small sample size, especially for patients with PND, hindered the exploration of the correlation between EIT parameters and biomarkers. Third, we did not categorized patients according to the severity of PND, which may be related to the degree of correlation between EIT parameters and biomarkers. Therefore, in future studies, we would like to include more patients and made the comparison between EIT and other cerebral monitoring modalities, including NIRS, EEG and TCD etc. Finally, randomized controlled intervention study using EIT information could be performed in the future.

In this study, we investigated the correlation between EIT extracted parameters during HCA period and the relative difference of neurological biomarkers level in serum. From physiological perspective view, we proved significant correlation between EIT information and the relative difference of biomarkers for patients with PND after TAAR. The evidence indicated that intraoperative EIT could provide auxiliary quantitative information for an early warning of brain injury during TAAR surgery. In addition, multiple validations of quantitative EIT with other techniques, including NIRS and TCD, have been studying by our group.

## Methods

### Study design and participants

**Figure 8:**
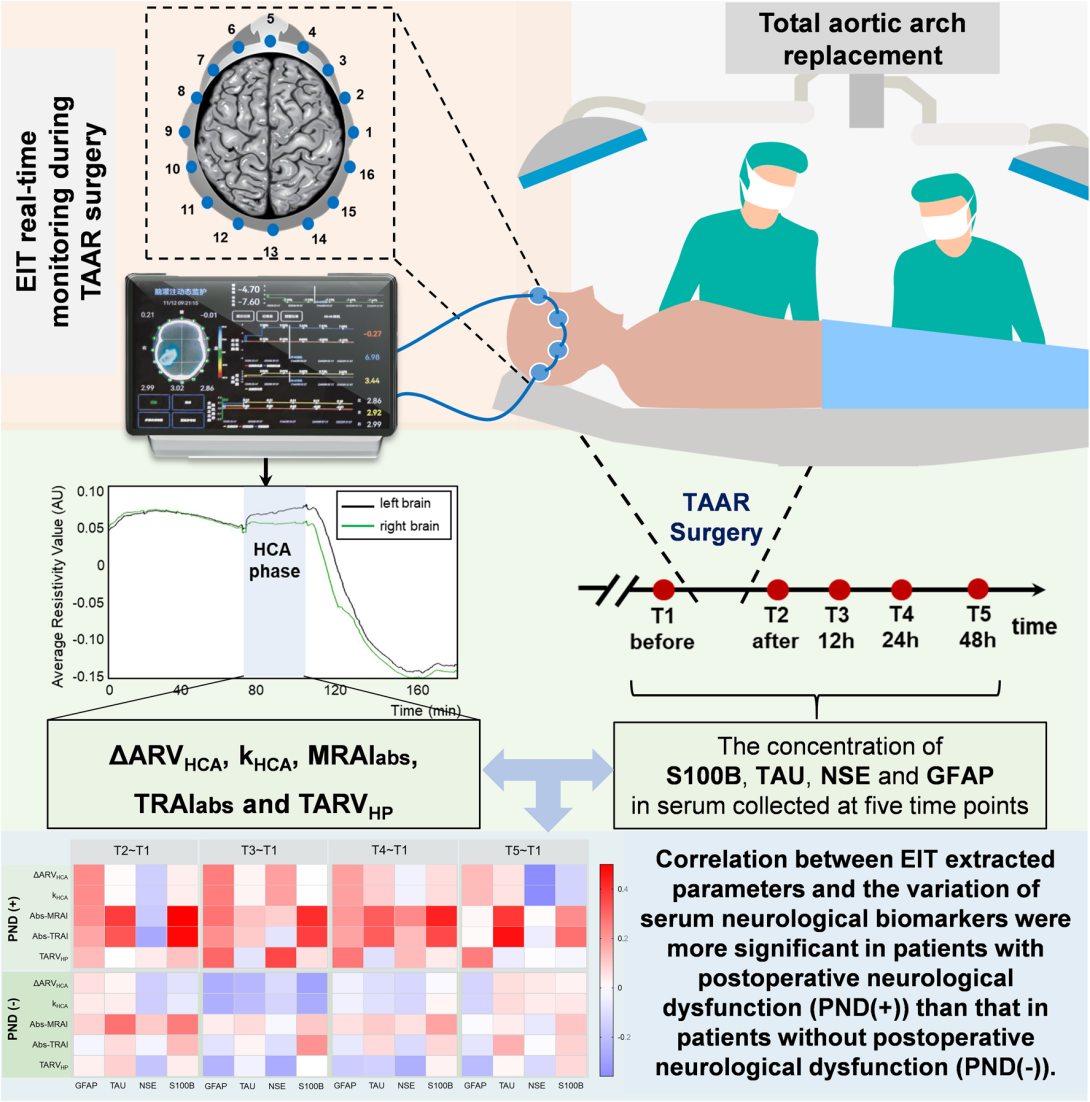
The correlation between EIT brain monitoring parameters during TAAR surgery and the variation of neurological biomarkers in serum were evaluated in order to explore the feasibility of brain EIT as an early warning assistant for brain injury.

We conducted a single-central prospective observational study. The primary objective of this trial was to evaluate the correlation between EIT signals obtained during TAAR surgery and the concentration of neurological biomarkers in serum before and after surgery (Fig. 8). This study was carried out in conformity with the Helsinki Declaration and was approved by the Medical Ethics Committee of Xijing Hospital of the Fourth Military Medical University (No. FMMU-E-III-001(1-7)). All subjects provided written informed consent. In addition, prior to enrollment, the trial was registered at Chinese Clinical Trial Registry (No. ChiCTR-OOC-16007844).

Patients included in the clinical trial satisfied the inclusion criteria included: (a) Stanford type A aortic dissection; (b) aged from 24 to 75, both male and female; (c) accepted for brain monitoring by EIT system (EH-300, China) during surgery; and (d) accepted for serum collection before and after surgery. In addition, the exclusion criteria: (a) not initial cardiac surgery or aortic stent implanted; (b) preoperative diagnosed with neurological injury; and (c) refused to participate the research.

### Intervention protocol and determination of postoperative neurological dysfunction

Intravenous anesthesia was supplemented with inhalation anesthesia, which included propofol, sevoflurane, sufentanil, dexmedetomidine and rocuronium at the beginning of anesthesia induction, and sufentanil, pipecuronium, midazolam and propofol were used for maintaining anesthesia during the operation. Postoperative analgesia was carried out with sufentanil and remifentanil. All surgical procedures were performed by the same surgical team.

Following heparinization, the patients received cardiopulmonary bypass (CPB), total body hypothermia, with cannulations of the right axillary and/or femoral arteries, superior and inferior vano cava. Cardiac arrest was induced with direct injections of high potassium cold blood cardioplegia into both coronary artery ostia. The aortic root was repaired with different techniques such as Bentall’s procedure, David’s procedure or Wheat’s procedure according to the situations. After cooling to target (25–26 °C for nasal pharyngeal and 26–28 °C for bladder) temperature, HCA was initiated, followed by unilateral or bilateral selective antegrade cerebral perfusion (SACP) via the right axillary artery and/or left common carotid artery. All of the surgeries adopted total arch replacement with frozen elephant trunk (FET) implantation. This procedure integrates total arch replacement using 4-branch arch Gelweave graft (Vascutek Terumo Inc, Scotland, England) and implantation of a frozen elephant trunk (MicroPort Medical, Shanghai, China) in the descending aorta as the treatment for extensive dissections or aneurysms involving the ascending aorta, the aortic arch, and the descending aorta. After anastomosing the 4-branch arch Gelweave graft and the proximal end of FET in the descending aortic, full CPB was restored with a cannulation to the perfusion branch of the 4-branch arch Gelweave graft and spontaneous heart rhythm resumed. Branches of the artificial vessel and left subclavian arteries, as well as the left common carotid artery, and innominate artery were anastomosed. When normal body temperature reached, CPB was weaned off, hemostasis and chest closure were carried out.

In the intensity care unit (ICU), the sedation status was determined using the Richmond agitation-sedation scale (RASS) for all patients and those with a RASS score of at least − 3 was necessary for performance of the confusion assessment method for the ICU (CAM-ICU) to determine if the patient was with delirium^40^. The CAM-ICU was conducted by a professional investigator three times per day during the first 72 h after ICU admission. Delirium of the patient was confirmed as present based either on a positive CAM-ICU score or on the administration of antipsychotic medications to treat delirium^40^. The diagnosis of neurological dysfunction was confirmed if the patient performed one or multiply clinical syndromes including delirium, seizures, coma lasting more than 24 h and new-onset stroke.

### Electrical impedance tomography data acquisition and definition

**Figure 9:**
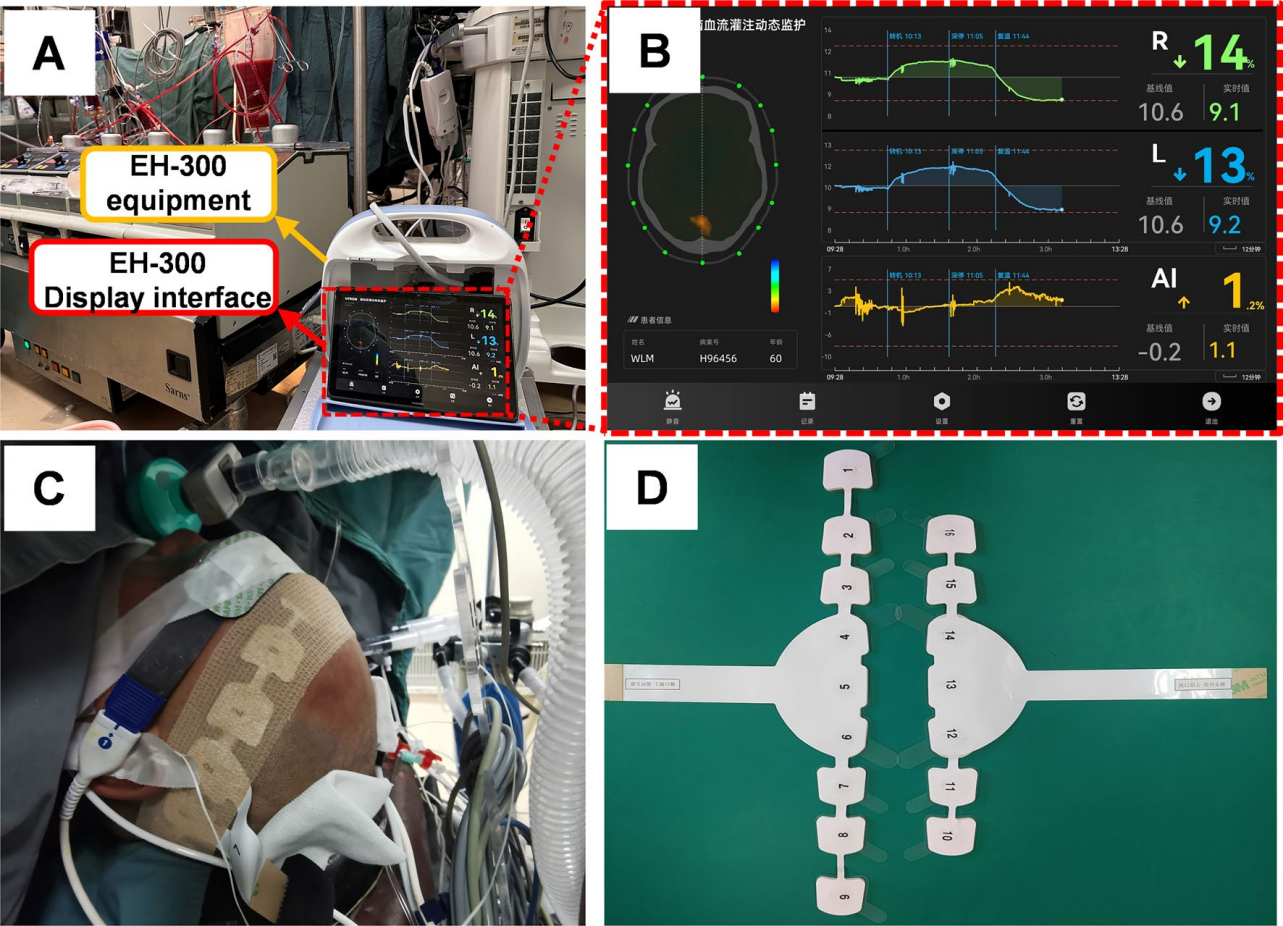
Set-up of EIT monitoring. (**A**) The set-up of EIT monitor during surgery. (**B**) The representative picture of EIT image captured during TAAR surgery. (**C**) The photo of electrodes placement captured during TAAR surgery. (**D**) The photo of electrodes. *EIT* electrical impedance tomography, *TAAR* total aortic arch replacement.

All patients were kindly asked to shave all their hair before surgery. An EIT equipment (EH-300, UTRON Technology Co., Ltd., Hangzhou, China) was used for brain EIT monitoring after aneasthesia (Fig. 9A,B). The EH-300 system used in this study was modified from a previous EIT monitoring system developed by our team in the early stage. The specifications of the EIT system are as follows: range of the working frequency, 1–190 kHz; measurement accuracy, ± 0.01%; and common mode rejection ratio, over 80 dB. Sixteen body surface electrodes, which are assembled into 2 disposable electrode tapes, were placed equidistantly on the surface of the cross-section above the patient’s ear (Fig. 9C,D). All the 16 electrodes were connected to the EIT machine. The patient’s cerebral impedance was monitored by the EIT system in real time. The opposite-drive adjacent-measurement protocol was used with a sinusoid current of 1 mAp-p at 50 kHz. The acquisition speed was 1 frame per second. The intraoperative data, which included clinical and EIT data, was recorded. The frame right before CPB period was selected as the reference frame and damped least-squares reconstruction algorithm was adopted for EIT imaging^41^.

Previous research showed that brain injury could appear in HCA phase due to the sudden change of oxygen supply to the brain^16^. Therefore, we focused on the EIT information around HCA phase to explore its correlation with above mentioned serum biomarkers. In order to extract significant information from electrical impedance signals, we chose to calculate five parameters based on average resistivity value (ARV) which was defined as:

1 $$\text{ARV}= \frac{1}{M}\sum {A}_{k}{x}_{k}, k=1,\dots , M,$$

where M is the total units of brain dissection model, ${\text{A}}_{k}$ is the area of kth unit and ${\text{x}}_{k}$ is the relative variation of electrical resistivity of the unit number $\text{k}$. ARV reflects the change of electrical impedance of the whole brain during surgery.

Therefore, the first parameter was defined as the difference of average resistivity value before and after HCA phase (${\Delta \text{ARV}}_{HCA}$):

2 $$\Delta {ARV}_{HCA}={ARV}_{after HCA}-{ARV}_{before HCA}.$$

${\Delta ARV}_{HCA}$ reflects the range of change of electrical impedance during HCA.

The second parameter was defined as:

3 $${k}_{HCA}=\frac{{\Delta ARV}_{HCA}}{{t}_{HCA}},$$

where ${k}_{HCA}$ stands for the slope of electrical impedance during HCA phase, and ${t}_{HCA}$ is the duration of HCA phase. ${k}_{HCA}$ represents for the speed of variation of electrical impedance during HCA.

Next, in order to analyze the difference behavior of left and right brain during HCA, we determined resistivity asymmetric index (RAI) between left and right cerebral electrical impedance, which was described as:

4 $$\text{RAI}= {ARV}_{L}-{ARV}_{R}.$$

Therefore, the third parameter was defined as the maximum value of the absolute resistivity asymmetric index (MAI_abs_).

The value of TRAI was described as:

5 $$\text{TRAI}=\int \limits_{start of HCA}^{end of HCA}RAI\cdot dt.$$

Since we adopted 1fps as the stimulating frequency, TRAI can be described as:

6 $$\text{TARI}=\sum_{HCA}{ARV}_{L}-{ARV}_{R}.$$

Therefore, the fourth was determined as the absolute value of the time integral of resistivity asymmetric index (TRAI_abs_). Finally, we also calculated the time integral of electrical impedance for half flow of perfusion (HP) as:

7 $${TARV}_{HP}=\sum_{HP}{ARV}_{HP}-{ARV}_{LP},$$

where ${ARV}_{HP}$ is for the ARV of half flow of perfusion and ${ARV}_{LP}$ is for the ARV of low flow perfusion. In fact, ${TARV}_{HP}$ revealed information including the variation of average brain electrical impedance from low to half flow perfusion and the duration of the variation.

### Blood sample collection and analysis

Equivalent blood samples for laboratory analysis were collected from the central venous catheter at five time points which were before surgery (T1), right after surgery (T2), 12 h after surgery (T3), 24 h after surgery (T4) and 48 h after surgery (T5) (Fig. 8). The sample tubes were centrifuged at 1400×*g* for 10 min to obtain the plasma and they were stored at − 80 °C for analysis. Neurological biomarkers GFAP, NSE, TAU and S100B were measured with commercial enzyme linked immunosorbent assay (ELISA) kits, which were GFAP ELISA kit (Abcam, cat, n. ab223867), NSE ELISA kit (Abcam, cat, n. ab217778), TAU ELISA kit (Abcam, cat, n. ab273617) and S100B ELISA kit (Abcam, cat, n. ab234573). The detection range was 0.31–30 ng/mL with a limit of detection of 0.04 ng/mL. For the non-detectable sample, the value was recorded as half of the limit of detection. The concentration of each biomarker was assessed. The relative difference of four biomarkers were determined as the variation between each value at each time point after surgery, which was T2, T3, T4 and T5, and that before surgery T1 then divided by the value at T1.

### Statistical analysis

Normal distribution was assessed by Shapiro–Wilk test. Relationship between five EIT parameters and relative difference of biomarkers were analyzed with Spearman or Pearson tests, depending on if both two groups of data were accord with normal distribution. Statistical significance was set at p < 0.05. In addition, we adopted the Benjamini and Hochberg method to apply the multiple testing correction. Calculations and statistical analysis were performed using MATLAB 2019b (MathWorks, Natick, USA), SPSS version 25.0 (SPSS Inc., Chicago, USA) and Prism 8.0 (GraphPad Software, La Jolla, CA).

### Supplementary Information

Supplementary Information.

## Data Availability

The related data and code can be obtained at https://osf.io/ucbd2/ after presenting request describing the intended use of the data.
