# Supplementary material for: Electrical impedance tomography provides information of brain injury during total aortic arch replacement through its correlation with relative difference of neurological biomarkers
Source: Sci Rep. 2024 Jun 20;14:14236. doi: 10.1038/s41598-024-65203-0 (PMC11190256; doi:10.1038/s41598-024-65203-0)
Supplement: Supplementary file 1 — Supplementary Information. [file 41598_2024_65203_MOESM1_ESM.pdf]

Supplementary file

Manuscript Title:

Electrical impedance tomography provides information of brain injury during total aortic arch replacement through its correlation with relative difference of neurological biomarkers

Authors:

Yitong Guo#, Chen Yang#, Wenjing Zhu#, Rong Zhao, Kai Ren, Weixun Duan, Jincheng Liu, Jing Ma, Xiuming Chen, Benyuan Liu, Canhua Xu, Zhenxiao Jin, Xuetao Shi.

#Yitong Guo, Chen Yang and Wenjing Zhu contributed equally to this work.

First corresponding: Xuetao Shi; Second corresponding: Zhenxiao Jin.

The correlation coefficients among five parameters were higher in patients with PND than those in patients without PND (Table S1-S3).

Table S1 Correlation coefficients for EIT extracted parameters for all patients. **means $p < 0.01$

	k_{HCA}	$MRAI_{abs}$	$TRAI_{abs}$	$TARV_{HP}$
ΔARV_{HCA}	0.995**	0.080	0.079	0.713**
k_{HCA}	...	0.063	0.066	0.716**
$MRAI_{abs}$	...	...	0.754**	-0.027
$TRAI_{abs}$	...	...	...	0.052

Table S2 Correlation coefficients for EIT extracted parameters for patients with PND.**means $p < 0.01$

	k_{HCA}	$MRAI_{abs}$	$TRAI_{abs}$	$TARV_{HP}$
ΔARV_{HCA}	1.000**	0.288	0.135	0.818**
k_{HCA}	...	0.288	0.135	0.818**
$MRAI_{abs}$	...	...	0.941**	0.159
$TRAI_{abs}$	...	...	...	-0.003

Table S3 Correlation coefficients for EIT extracted parameters for patients without PND.**means $p < 0.01$

	k_{HCA}	$MRAI_{abs}$	$TRAI_{abs}$	$TARV_{HP}$
ΔARV_{HCA}	0.992**	0.033	0.086	0.684**
k_{HCA}	...	0.016	0.061	0.698**
$MRAI_{abs}$	...	...	0.689**	-0.075
$TRAI_{abs}$	...	...	...	0.071

The correlation coefficients among four biomarkers' area under curve in serum for five time points were significantly greater in patients with PND than those in patients without PND (Table S4-S6). Moreover, the concentrations of biomarkers in serum for each time point were showed in Table S7. Both the concentrations of TAU protein before surgery and right after surgery were significantly higher in PND (+) than those in PND (-) (Table S7). In addition, concentrations of GFAP 24h and 48h after surgery were significantly higher in PND (+) than those in PND (-) (600.05 (386.25~ 1654.76) vs. 411.82 (327.52~ 503.07) and 716.71 (391.24~ 1429.33) vs. 402.56 (319.75~ 554.14), respectively) (Table S7). Unfortunately, after applying the multiple testing correction procedure, there was no significant difference between two groups (with α equal to 0.05). In addition, the relative difference of neurological biomarkers in serum before and after surgery were obtained by calculation (Table S8). Results showed that only the variation between T2 and T1 for S100B protein was significantly different between PND (+) and PND (-) (0.57 (-0.33~3.3) vs. -0.11 (-0.41~0.37), P=0.041) (Table S8). However, after applying the multiple testing correction procedure, there was no significant difference between two groups (with α equal to 0.05).

Table S4 Correlation coefficients of the area under curve (AUC) for concentration of each biomarker in serum for all patients.

	AUC-TAU	AUC-GFAP	AUC-NSE
AUC-S100B	0.746**	0.505**	0.437**
AUC-TAU	...	0.506**	0.467**
AUC-GFAP	...	...	0.492**

**means p < 0.01.

Table S5 Correlation coefficients of the area under curve (AUC) for concentration of each biomarker in serum for patients with PND.

	AUC-TAU	AUC-GFAP	AUC-NSE
AUC-S100B	0.865**	0.785**	0.718**
AUC-TAU	...	0.600**	0.744**
AUC-GFAP	...	...	0.556**

**means p < 0.01 and *means p < 0.05.

Table S6 Correlation coefficients of the area under curve (AUC) for concentration of each biomarker in serum for patients without PND.

	AUC-TAU	AUC-GFAP	AUC-NSE
AUC-S100B	0.649**	0.349*	0.242
AUC-TAU	…	0.420**	0.286
AUC-GFAP	…	…	0.387*

**means $p < 0.01$ and *means $p < 0.05$.

Table S7 Concentration (pg/mL) of neurological biomarkers in serum before and after TAAR surgery for patients with and without postoperative neurological dysfunction (PND (+) and PND (-)).

**Biomarkers**	**PND (+) N=16**	**PND (-) N=41**	***P* value**
S100B-T1	59.89 (16.45~ 120.71)	32.63 (19.23~ 76.81)	0.303
S100B-T2	96.24 (32.16~ 212.57)	33.94 (22.6~ 78.19)	0.057
S100B-T3	60.77 (28.08~ 165.55)	29.7 (16.53~ 66.13)	0.062
S100B-T4	57.77 (26.8~ 115.87)	35.15 (15.16~ 82.78)	0.171
S100B-T5	34.65 (24.1~ 94.27)	34.06 (19.38~ 74.92)	0.763
TAU-T1	99.88 (50.58~ 353.66)	61.10 (34.22~ 84.66)	0.036*
TAU-T2	150.37 (80.74~ 375.35)	63.29 (33.14~ 113.72)	0.020*
TAU-T3	88.36 (51.55~ 307.81)	53.58 (27.25~ 108.23)	0.065
TAU-T4	70.04 (46.07~ 328.40)	51.96 (26.77~ 92.19)	0.118
TAU-T5	76.75 (38.58~ 185.90)	44.52 (34.78~ 81.50)	0.145
GFAP-T1	476.06 (296.42~ 1581.96)	430.34 (300.12~ 527.70)	0.279
GFAP-T2	603.97 (343.31~ 1280.98)	492.97 (363.03~ 678.95)	0.279
GFAP-T3	481.66 (380.74~ 2141.48)	406.50 (323.20~ 583.89)	0.062
GFAP-T4	600.05 (386.25~ 1654.76)	411.82 (327.52~ 503.07)	0.030*
GFAP-T5	716.71 (391.24~ 1429.33)	402.56 (319.75~ 554.14)	0.013*
NSE-T1	792.08 (289.71~ 3039.09)	456.56 (196.66~ 1402.69)	0.263
NSE-T2	1340.18 (843.84~ 4913.65)	1640.98 (890.86~ 3490.67)	0.958
NSE-T3	1682.33 (548.40~ 3269.67)	1378.88 (735.55~ 2633.69)	0.972
NSE-T4	1993.33 (934.76~ 4074.38)	1097.64 (590.21~ 2528.46)	0.145
NSE-T5	1696.36 (620.57~ 4613.69)	1116.06 (504.25~ 2079.65)	0.183

Data are presented as median (IQR). T1: time before surgery; T2: time right after surgery; T3: 12h after surgery; T4: 24h after surgery; T5: 48h after surgery; * *P* value < 0.05 means the value was significantly different between PND(+) and PND(-).

Table S8 Relative difference of neurological biomarkers in serum before and after TAAR surgery for patients with and without postoperative neurological dysfunction (PND (+) and PND (-)).

**RF of biomarkers**	**PND (+) N=16**	**PND (-) N=41**	***P* value**
S100B-T2T1	0.57 (-0.33~ 3.3)	-0.11 (-0.41~ 0.37)	0.041*
S100B-T3T1	0.06 (-0.42~ 0.48)	0.00 (-0.51~ 0.67)	0.859
S100B-T4T1	0.05 (-0.49~ 0.78)	-0.11 (-0.53~ 1.75)	0.749
S100B-T5T1	-0.26 (-0.69~ 1.06)	0.07 (-0.63~ 1.41)	0.776
TAU-T2T1	0.39 ± 0.79	0.07 (-0.31~ 1.14)	0.109
TAU-T3T1	0.15 ± 0.56	-0.11 (-0.38~ 0.69)	0.163
TAU-T4T1	0.01 ± 0.69	-0.25 (-0.56~ 0.88)	0.084
TAU-T5T1	0.05 ± 0.75	-0.28 (-0.58~ 1.18)	0.061
GFAP-T2T1	0.09 (-0.13~ 0.25)	0.16 (-0.17~ 0.61)	0.477
GFAP-T3T1	0.10 (-0.14~ 0.82)	0.10 (-0.21~ 0.42)	0.790
GFAP-T4T1	0.05 (-0.21~ 0.37)	0.00 (-0.25~ 0.30)	0.394
GFAP-T5T1	0.19 (-0.25~ 1.53)	-0.04 (-0.30~ 0.44)	0.365
NSE-T2T1	1.92 ± 2.16	1.52 (0.27~ 5.16)	0.657
NSE-T3T1	0.86 (-0.17~ 2.18)	1.44 (-0.06~ 4.77)	0.375
NSE-T4T1	2.11 (-0.12~ 3.92)	1.19 (-0.23~ 4.53)	0.776
NSE-T5T1	1.21 (-0.15~ 3.48)	1.23 (-0.13~ 3.88)	0.817

Data are presented as median (IQR) or mean±SD. RF: relative difference; T2T1: the relative difference between after and before surgery; T3T1: relative difference between 12h after and before surgery; T4T1: relative difference between 24h after and before surgery; T5T1: relative difference between 48h after and before surgery; * P value < 0.05 means the value was significantly different between PND(+) and PND(-).

Table S9 Correlation coefficients between relative difference of serum biomarkers and EIT extracted parameters for patients with and without postoperative neurological dysfunction (PND).

		T2~T1				T3~T1				T4~T1				T5~T1			
		GFAP	TAU	NSE	S100B	GFAP	TAU	NSE	S100B	GFAP	TAU	NSE	S100B	GFAP	TAU	NSE	S100B
PND (+)	ΔARV_{HCA}	0.226	0.006	-0.156	0.032	0.253	0.021	0.188	-0.006	0.185	0.094	-0.041	0.065	0.135	0.056	-0.359	-0.141
	k_{HCA}	0.221	0.382	-0.171	**0.500***	0.274	0.121	0.094	0.412	0.200	0.318	0.232	0.435	0.015	0.391	0.006	0.224
	$MRAI_{abs}$	0.176	0.332	-0.274	**0.485***	0.218	0.103	-0.088	0.376	0.182	0.318	0.132	0.356	0.009	0.468	-0.059	0.279
	$TRAI_{abs}$	0.135	-0.003	0.038	0.118	0.297	-0.088	0.359	0.059	0.265	-0.100	0.135	0.038	0.256	-0.059	-0.009	-0.029
	$TARV_{HP}$	0.061	0.021	-0.155	-0.092	-0.257	-0.229	-0.119	-0.294	-0.048	-0.105	-0.128	-0.019	-0.136	0.064	0.045	0.024
PND (-)	ΔARV_{HCA}	0.04	0.036	-0.160	-0.067	-0.252	-0.214	-0.138	-0.264	-0.049	-0.098	-0.13	0.019	-0.135	0.069	0.038	0.046
	k_{HCA}	0.087	0.276	0.102	0.256	0.036	0.070	-0.027	0.106	0.061	0.102	0.057	0.179	-0.014	0.034	-0.074	0.112
	$MRAI_{abs}$	-0.034	0.068	-0.036	0.135	-0.090	0.020	-0.104	0.213	-0.073	0.071	-0.119	0.077	-0.083	0.155	-0.011	0.088
	$TRAI_{abs}$	0.029	0.092	-0.188	0.036	-0.24	-0.045	-0.266	-0.003	-0.163	-0.147	-0.254	0.025	-0.217	0.017	-0.121	0.074
	$TARV_{HP}$	0.226	0.006	-0.156	0.032	0.253	0.021	0.188	-0.006	0.185	0.094	-0.041	0.065	0.135	0.056	-0.359	-0.141

*means $p < 0.05$. T2~T1: relative difference between after and before surgery; T3~T1: relative difference between 12h after and before surgery; T4~T1: relative difference between 24h after and before surgery; T5~T1: relative difference between 48h after and before surgery; HCA: hypothermic circulatory arrest; ΔARV_{HCA}: the difference of average resistivity value before and after HCA phase; k_{HCA}: the slope of electrical impedance during HCA phase; $MRAI_{abs}$: maximum of the absolute value of resistivity asymmetric index; $TARI_{abs}$: absolute value of time integral of resistivity asymmetric index; $TARV_{HP}$: time integral of electrical impedance for half flow of perfusion.

Table S10. Correlation coefficients between relative difference of serum biomarkers and EIT extracted parameters for patients with normal and abnormal concentration of S100B before surgery.

		T2~T1	T3~T1	T4~T1	T5~T1
PND (+)	ΔARV_{HCA}	-0.134	-0.230	-0.112	0.004
	k_{HCA}	-0.119	-0.203	-0.076	0.026
	$MRAI_{abs}$	0.344	0.185	0.241	0.208
	$TRAI_{abs}$	0.227	0.286	0.144	0.268
	$TARV_{HP}$	0.051	0.048	-0.058	0.031
PND (-)	ΔARV_{HCA}	-0.018	0.055	**0.758***	-0.018
	k_{HCA}	-0.018	0.055	**0.758***	-0.018
	$MRAI_{abs}$	0.248	0.467	**0.743***	-0.248
	$TRAI_{abs}$	0.184	0.447	0.300	-0.079
	$TARV_{HP}$	-0.018	0.091	0.491	0.127

*means $p < 0.05$. T2~T1: relative difference between after and before surgery; T3~T1: relative difference between 12h after and before surgery; T4~T1: relative difference between 24h after and before surgery; T5~T1: relative difference between 48h after and before surgery; HCA: hypothermic circulatory arrest; ΔARV_{HCA}: the difference of average resistivity value before and after HCA phase; k_{HCA}: the slope of electrical impedance during HCA phase; $MRAI_{abs}$: maximum of the absolute value of resistivity asymmetric index; $TARI_{abs}$: absolute value of time integral of resistivity asymmetric index; $TARV_{HP}$: time integral of electrical impedance for half flow of perfusion.

Table S11. Correlation coefficients between relative difference of serum biomarkers and EIT extracted parameters for patients with normal and abnormal concentration of TAU before surgery.

		T2~T1	T3~T1	T4~T1	T5~T1
PND (+)	ΔARV_{HCA}	-0.079	-0.261	-0.145	0.091
	k_{HCA}	-0.073	-0.240	-0.138	0.099
	$MRAI_{abs}$	0.226	0.031	0.130	0.106
	$TRAI_{abs}$	0.103	-0.044	0.0480	0.210
	$TARV_{HP}$	-0.001	-0.085	-0.176	0.041
PND (-)	ΔARV_{HCA}	**0.770****	0.394	0.539	0.091
	k_{HCA}	**0.794****	0.455	0.515	0.127
	$MRAI_{abs}$	0.079	0.345	0.176	0.212
	$TRAI_{abs}$	-0.103	0.176	0.018	0.248
	$TARV_{HP}$	**0.818****	0.091	0.200	-0.200

**means $p < 0.01$. T2~T1: relative difference between after and before surgery; T3~T1: relative difference between 12h after and before surgery; T4~T1: relative difference between 24h after and before surgery; T5~T1: relative difference between 48h after and before surgery; HCA: hypothermic circulatory arrest; ΔARV_{HCA}: the difference of average resistivity value before and after HCA phase; k_{HCA}: the slope of electrical impedance during HCA phase; $MRAI_{abs}$: maximum of the absolute value of resistivity asymmetric index; $TARI_{abs}$: absolute value of time integral of resistivity asymmetric index; $TARV_{HP}$: time integral of electrical impedance for half flow of perfusion.

Table S12. Correlation coefficients between relative difference of serum biomarkers and EIT extracted parameters for patients with normal and abnormal concentration of GFAP before surgery.

		T2~T1	T3~T1	T4~T1	T5~T1
PND (+)	ΔARV_{HCA}	0.232	0.147	0.268	-0.315
	k_{HCA}	0.226	0.224	0.312	-0.226
	$MRAI_{abs}$	-0.338	0.003	0.015	0.015
	$TRAI_{abs}$	0.112	**0.521***	**0.521***	0.321
	$TARV_{HP}$	0.306	0.285	0.179	-0.153
PND (-)	ΔARV_{HCA}	0.084	-0.203	-0.018	-0.035
	k_{HCA}	0.060	-0.214	-0.032	-0.043
	$MRAI_{abs}$	0.230	0.102	0.111	-0.078
	$TRAI_{abs}$	-0.001	-0.127	-0.102	-0.103
	$TARV_{HP}$	-0.042	-0.185	-0.113	-0.086

*means $p < 0.05$. T2~T1: relative difference between after and before surgery; T3~T1: relative difference between 12h after and before surgery; T4~T1: relative difference between 24h after and before surgery; T5~T1: relative difference between 48h after and before surgery; HCA: hypothermic circulatory arrest; ΔARV_{HCA}: the difference of average resistivity value before and after HCA phase; k_{HCA}: the slope of electrical impedance during HCA phase; $MRAI_{abs}$: maximum of the absolute value of resistivity asymmetric index; $TARI_{abs}$: absolute value of time integral of resistivity asymmetric index; $TARV_{HP}$: time integral of electrical impedance for half flow of perfusion.

Table S13. Correlation coefficients between relative difference of serum biomarkers and EIT extracted parameters for patients with normal and abnormal concentration of NSE before surgery.

	T2~T1	T3~T1	T4~T1	T5~T1
ΔARV_{HCA}	-0.100	0.043	-0.017	0.013
k_{HCA}	-0.099	0.039	-0.015	0.020
$MRAI_{abs}$	-0.016	0.026	0.098	-0.045
$TRAI_{abs}$	-0.083	-0.027	0.019	0.023
$TARV_{HP}$	-0.029	0.036	-0.059	-0.034

T2~T1: relative difference between after and before surgery; T3~T1: relative difference between 12h after and before surgery; T4~T1: relative difference between 24h after and before surgery; T5~T1: relative difference between 48h after and before surgery; HCA: hypothermic circulatory arrest; ΔARV_{HCA}: the difference of average resistivity value before and after HCA phase; k_{HCA}: the slope of electrical impedance during HCA phase; $MRAI_{abs}$: maximum of the absolute value of resistivity asymmetric index; $TARI_{abs}$: absolute value of time integral of resistivity asymmetric index; $TARV_{HP}$: time integral of electrical impedance for half flow of perfusion.
